# β-arrestin1 protects intestinal tight junction through promoting mitofusin 2 transcription to drive parkin-dependent mitophagy in colitis

**DOI:** 10.1093/gastro/goae084

**Published:** 2024-09-06

**Authors:** Shuyun Wu, Huiling Liu, Jiazhi Yi, Minyi Xu, Jie Jiang, Jin Tao, Bin Wu

**Affiliations:** Department of Gastroenterology, The Third Affiliated Hospital of Sun Yat-Sen University, Guangzhou, Guangdong, P. R. China; Department of Gastroenterology, The Third Affiliated Hospital of Sun Yat-Sen University, Guangzhou, Guangdong, P. R. China; Department of Gastroenterology, Zhujiang Hospital, Southern Medical University, Guangzhou, Guangdong, P. R. China; Department of Gastroenterology, The Third Affiliated Hospital of Sun Yat-Sen University, Guangzhou, Guangdong, P. R. China; Department of Gastroenterology, The Third Affiliated Hospital of Sun Yat-Sen University, Guangzhou, Guangdong, P. R. China; Department of Gastroenterology, The Third Affiliated Hospital of Sun Yat-Sen University, Guangzhou, Guangdong, P. R. China; Department of Gastroenterology, The Third Affiliated Hospital of Sun Yat-Sen University, Guangzhou, Guangdong, P. R. China

**Keywords:** β-arrestin1, mitochondrial dysfunction, mitophagy, mitofusion2, intestinal barrier

## Abstract

**Background:**

Intestinal barrier defect is an essential inflammatory bowel disease (IBD) pathogenesis. Mitochondrial dysfunction results in energy deficiency and oxidative stress, which contribute to the pathogenesis of IBD. β-arrestin1 (ARRB1) is a negative regulator that promotes G protein-coupled receptors desensitization, endocytosis, and degradation. However, its role in maintaining the intestinal barrier remains unclear.

**Methods:**

Dextran sulfate sodium-induced colitis was performed in ARRB1 knockout and wild-type mice. Intestinal permeability and tight junction proteins were measured to evaluate the intestinal barrier. Mitochondria function and mitophagic flux in mice and cell lines were detected. Finally, the interaction between ARRB1 and mitofusin 2 was investigated by co-immunoprecipitation and dual luciferase assay.

**Results:**

We identified that ARRB1 protected the intestinal tight junction barrier against experimental colitis *in vivo*. ARRB1 deficiency was accompanied by abnormal mitochondrial morphology, lower adenosine triphosphate (ATP) production, and severe oxidative stress. *In vitro*, the knockdown of ARRB1 reduced ATP levels and mitochondrial membrane potential while increasing reactive oxygen species levels and oxidative stress. Upon ARRB1 ablation, mitophagy was inhibited, accompanied by decreased LC3BII, phosphatase and tension homologue-induced protein kinase1 (PINK1), and parkin, but increased p62 expression. Mitophagy inhibition via PINK1 siRNA or mitochondrial division inhibitor 1 impaired ARRB1-mediated tight junction protection. The interaction of ARRB1 with E2F1 activated mitophagy by enhancing the transcription of mitofusin 2.

**Conclusions:**

Our results suggest that ARRB1 is critical to maintaining the intestinal tight junction barrier by promoting mitophagy. These results reveal a novel link between ARRB1 and the intestinal tight junction barrier, which provides theoretical support for colitis treatment.

## Graphical Abstract

**Graphical Abstract:**
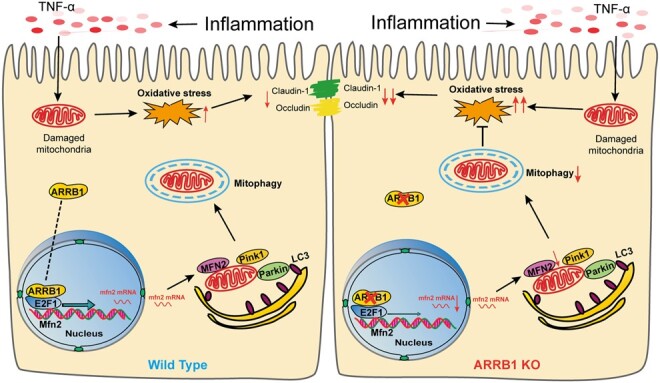

## Introduction

Inflammatory bowel disease (IBD), including Crohn’s disease (CD) and ulcerative colitis (UC), is a multifactorial disease involving immune dysregulation, genetic susceptibility, environmental factors, and microbial dysbiosis [1]. The intestinal epithelial barrier defends against para-cellular permeation of endogenous and exogenous noxious antigens and pathogens in the intestinal lumen. Accumulating evidence indicates that the loss of intestinal barrier integrity contributes to increased intestinal permeability, triggering or accelerating intestinal inflammation [2, 3]. The intestinal barrier comprises several cellular components, including tight junctions, adherence junctions, and desmosomes [4]. Tight junctions limit the para-cellular permeability of luminal proinflammatory molecules [5]. Increased intestinal permeability has been observed in IBD, accompanied by abnormal tight junctions structure and a down-regulation of several tight junctions proteins, including claudins and occludin [6]. Recent research has demonstrated that regulation of tight junctions integrity ameliorates intestinal inflammation [7, 8]. Thus, protecting epithelial barrier function could be a potential therapeutic strategy for IBD.

Oxidative stress is essential in the pathogenesis of gastrointestinal mucosal diseases [9]. Mitochondria are the primary source of intracellular reactive oxygen species (ROS). Since maintaining intestinal epithelial cells junction integrity is energy-dependent, mitochondrial function might be central to the appropriate preservation of epithelial barrier function. It has been shown that the potential factors that induce mitochondrial dysfunction contribute to IBD susceptibility [10, 11]. Mitochondrial abnormal structure and dysfunction have been observed in patients with IBD [12], and mitochondrial ROS and mitochondrial DNA from damaged mitochondria are proinflammatory factors [13]. Mitophagy is a necessary and specific form of autophagy that selectively removes dysfunctional or redundant mitochondria [14]. Mitophagy has been demonstrated to have a neuroprotection effect in Alzheimer’s disease [15, 16] and be involved in innate immunity [17, 18]. Compromised mitophagy has further been observed in functional studies of CD-associated risk variants in autophagy-related ATG16L1 and immunity-related GTPase M [19]. Preliminary studies indicate mitophagy as a protective process in IBD by promoting proinflammatory cytokine production, intestinal epithelial cell viability, and possibly pathogen clearance [20, 21]. Furthermore, mitophagy has become integral to immune cell development, activation, and differentiation [22]. Thus, maintaining mitophagy might play a uniquely protective role in preventing the disease progression of IBD.

β-arrestin1 (ARRB1) is a negative G protein-coupled receptor (GPCR) signaling regulator. It promotes GPCR desensitization, endocytosis, and degradation [23]. ARRB1 has also been demonstrated as a molecular scaffold that regulates cellular function through interactions with other partner proteins and contributes to immune response, inflammation, and tumorigenesis [24]. Mounting evidence also suggests that ARRB1 can translocate from the cytoplasm to the nucleus and modulate targeted gene transcription [25]. Nuclear ARRB1 interacts with transcription co-factors, such as p300 and IκBα, to play a vital role in cell growth, apoptosis, and immune function [26, 27]. ARRB1 has been identified as a critical regulator of glucose and energy homeostasis [28]. Pan *et al*. [29] found that high glucose attenuated the cardioprotective effects of glucagon-like peptide 1 through induction of mitochondrial dysfunction via inhibition of ARRB1. Nevertheless, ARRB1 has increased mitochondrial oxidative stress in cultured human cardiac fibroblasts [30]. Hence, the role of ARRB1 in mitochondrial function requires further investigation.

The role of ARRB1 in IBD is still controversial. A previous study found the deficiency of ARRB1 protects against experimental colitis [31, 32]. However, we previously demonstrated that ARRB1 mediated mucosal protection by COX-1/PGE2/EP4 in colitis [33]. Furthermore, it has not yet been established whether ARRB1 regulates epithelial barrier function in colitis. Our study demonstrated that ARRB1 deficiency impaired the intestinal tight junctions barrier through mitochondrial dysfunction accompanied by lower adenosine triphosphate (ATP) production and increased oxidative stress in colitis mice. Mechanistically, ARRB1 enhanced mitophagy to maintain mitochondrial function through activating mitofusin 2 (MFN2) transcription. These results reveal a novel link between ARRB1 and the intestinal tight junction barrier in colitis, which provides theoretical support for colitis treatment.

## Materials and methods

### Mice

All animal procedures were approved by the Research Ethics Committee of the Third Affiliated Hospital of Sun Yat-Sen University (IACUC-F3-20-0911). C57BL/6 genetic background mice were used. The original Arrb1 heterozygous mice were kindly provided by Dr R. J. Lefkowitz from Duke University Medical Center (Durham, NC, USA). All colonies were housed in micro isolator cages with 50% humidity and 12-h light-dark cycles. Six- to eight-week-old and sex-matched mice were randomly assigned to groups. Three percent dextran sulfate sodium (DSS; MP Biomedicals LLC, Solon, OH, USA) in drinking water for 7 days was administered to induce acute colitis in mice.

### Cell culture and treatment

The HCoEpic cells were initially obtained from Sciencell Research Laboratories (Carlsbad, CA, USA). The HCoEpic cells were cultured in DMEM medium containing 10% fetal bovine serum (CellCook, Guangzhou, Guangdong, China) at 37°C under 5% CO_2_. For drug intervention, cells were treated with 40 ng/μL TNF-α (PeproTech, Hamburg, Germany), indicated concentration of 10 μM carbonyl cyanide 3-chlorophenylhydrazone (CCCP), 10 μM mitochondrial division inhibitor 1 (Mdivi-1), 10 μM N-acetylcysteine (NAC), 10 μM 3-Methyladenine (3-MA), and 50 μM Chloroquine (CQ) from MedChemExpress (Monmouth Junction, NJ, USA).

### Transient transfection, stable cell line generation, and RNA interference

Transient transfection was performed according to the manufacturer’s protocol (jetPRIME transfection agents; Polyplus, Illkirch, France). Small interfering RNAs, including si-mfn2 (5ʹ-ACUUUGUCACUGCCAAGAA-3′ʹ), si-pink1 (5ʹ-ACUUUGUCACUG-CCAAGAA-3ʹ), si-E2F1 (5ʹ-GUCACGCUAUGAGACCU-CA-3ʹ), were used according to the manufacturer’s instructions (Gene Pharma, Shanghai, China).

pLenti-ARRB1-puromycin (GeneChem, Shanghai, China) was used for lentiviral transfection to generate the stable *ARRB1*-overexpression cell line. *ARRB1*-shRNA was cloned into the lentiviral vector GV112 (GeneChem) to generate the stable *ARRB1* knockdown cell line. Stable transfections were selected with respective antibiotics for 2 weeks.

### Plasmid transfection

Briefly, 70%–80% of confluent cells were transfected. Lipofectamine 3000 reagent (Invitrogen, Waltham, MA, USA) was used to deliver plasmid DNAs into cells growing in serum-free opti-MEM media. Subsequent experiments were completed 24 h after transfection. The GFP-LC3 and parkin plasmids were kindly provided by Professor Yunfei Qin (Department of The Biological Therapy Center, The Third Affiliated Hospital of Sun Yat-Sen University, Guangzhou, China). ARRB1 plasmid was kindly provided by Dr Gan Pei (Chinese Academy of Sciences, Shanghai, China). PINK1, MFN2, E2F1, ARRB1 (Q394L), and ARRB1 (1–163) plasmids were created and synthesized by YouminBio (Guangzhou, Guangdong, China). The pcDNA3.0-*Vector* was transfected as the negative control.

### H&E, immunohistochemistry, and immunofluorescence staining

As previously described, hematoxylin and eosin (H&E), immunohistochemistry, and immunofluorescence staining were performed [33]. After antigen retrieval was performed, 3-μm-thick paraffin-embedded sections were incubated with the indicated primary antibodies against MFN2 (1:200; No. 12186-1-AP; Proteintech, Rosemont, IL, USA), cytochrome c oxidase IV (COX IV) (1:200; No. 11242-1-AP; Proteintech), claudin 1 (1:200; No. ab15098; Abcam, Cambridge, MA, USA), and occludin (1:200; No. 27260-1-AP; Proteintech) overnight at 4°C. For immunohistochemistry, the sections were incubated with secondary antimouse/rabbit IgG (1:300; No. A0208/A0216; Beyotime, Shanghai, China) at 37°C for 2 h and then stained with diaminobenzidine and hematoxylin. For immunofluorescence staining in tissue samples, the sections were incubated with the related biotin-conjugated secondary antibody and streptavidin Alexa Fluor 488 or 594 (1:300; No. A-11001/No. A-11012; Invitrogen) at 37°C for 2 h. 4,6-diamidino-2-phenylindole (DAPI; No. D1306; Invitrogen) was used to stain nuclei. For immunofluorescence staining in cells, cells were fixed with 4% paraformaldehyde for 20 min at room temperature and incubated with 0.5% Triton X-100 (No. ST797; Beyotime). After washing with phosphate-buffered saline (PBS) three times, cells were incubated with primary antibodies against COX IV (1:200; No. 11242-1-AP; Proteintech) overnight at 4°C, followed by the corresponding biotin-conjugated secondary antibody and streptavidin Alexa Fluor 488 or 594 at 37°C for 2 h. DAPI was used to stain nuclei.

### Quantitative RT-PCR

Total RNA was extracted using TRIzol (No. 15596–018; Invitrogen) and transcribed into cDNA using a High Capacity cDNA Kit (No. FSQ101; TOYOBO, Japan). Then aliquots of cDNA were amplified using gene-specific primers and ChamQ SYBR qPCR Master Mix (No. Q441; Vazyme, Nanjing, Jiangsu, China) in a real-time PCR system (Bio-Rad, Hercules, CA, USA). Each sample was tested in triplicate. Relative expression levels were calculated by the 2^−ΔΔCt^ method and normalized to β-actin. The sequences of the primers are listed in Supplementary Table 1.

### Western-blot analysis and co-immunoprecipitation

Total protein was either isolated from the intestinal tissues or cells using a lysis buffer supplemented with a protease and phosphatase inhibitor cocktail (No. 78430; Thermo Scientific, Waltham, MA, USA). Immunoblotting was performed as described previously [33]. Western blotting was performed using antibodies against ARRB1 (1:1000; No. ab32099; Abcam), SQSTM1/p62 (1:1000; No. 18420-1-AP; Proteintech), LC3B (1:1000; No. L7543; Sigma-Aldrich, St Louis, MO, USA), PINK1 (1:1000; No. 23274-1-AP; Proteintech), parkin (1:1000; No. 14060-1-AP; Proteintech), claudin 1 (1:1000; No. ab15098; Abcam), occludin (1:1000; No. 27260-1-AP; Proteintech), MFN2 (1:1000; No. 12186-1-AP; Proteintech), COX IV (1:1000; No. 11242-1-AP; Proteintech), E2F1 (1:100; No. sc-251; Santa Cruz, CA, USA), and GAPDH (1:5000; No. 2118S; Cell Signaling Technology, Danvers, MA, USA). Polyvinylidene fluoride membranes with proteins were incubated overnight with primary antibodies at 4°C and corresponding secondary antibodies at room temperature for 2 h. Proteins were visualized using a Bio-Rad ECL machine. The protein bands were quantified using ImageJ software (US National Institutes of Health, Bethesda, MD, USA).

For immunoprecipitation, cells were lysed in IP Lysis Buffer (No. P0013; Beyotime) with a protease and phosphatase inhibitor cocktail (No. 78430; Thermo Scientific). The lysate was precleared with protein A magnetic beads (No. 1614013; Bio-Rad) at 4°C overnight and then incubated with anti-ARRB1 and anti-E2F1 antibodies for 2 h. Antimouse or rabbit IgG antibodies (No. A7016/No. A7028; Beyotime) from the related species were used as a control. After removing the beads by centrifugation, the boiled samples were subjected to immunoblot analysis.

### Cell viability assay

Cell counting kit-8 (CCK-8; Dojindo Laboratories, Kumamoto, Japan) assays were used to measure cell viability. HCoEpic cells (2.5 × 10^3^/well) were seeded into 96-well plates. After treatment with the indicated chemicals, 10 μL CCK-8/90 μL culture medium was added to each well. After incubation at 37°C for 2 h, the absorbance at 450 nm was measured by a spectrophotometer (BioTek-Epoch2; Santa Clara, CA, USA). The experiments were performed in triplicate wells and three times independently.

### ATP measurement

The ATP content was measured using the ATP assay kit (No. S0027; Beyotime). Cells or tissue samples were washed with ice-cold PBS and then homogenized and sonicated in lysis buffer on ice. After sonication, the lysed cells or tissues were centrifuged at 12,000×*g* for 5 min to remove debris. Then, ATP was determined using the ATP assay kit based on the luciferin/luciferase assay and normalized for protein content.

### Intracellular ROS detection

Intracellular ROS levels were measured using an oxidation-sensitive fluorescent probe, 2′-7′dichlorofluorescin diacetate (DCFH-DA). Cells were washed twice in PBS and incubated with 10 μM DCFH-DA at 37°C for 30 min. DCFH-DA was deacetylated intracellularly by nonspecific esterases and further oxidized ROS to the fluorescent compound 2,7-dichlorofluorescein (DCF). DCF fluorescence was detected using flow cytometry (excitation wavelength at 488 nm and emission wavelength at 525 nm).

### Measurement of malondialdehyde and glutathione

The malondialdehyde and glutathione levels were measured according to the manufacturer’s protocol (Boxbio Science & Technology, Beijing, China). Malondialdehyde measurement was determined by the reaction of thiobarbituric acid (TBA) with malondialdehyde to generate the stable end product of the malondialdehyde-TBA adduct. The cells were lysed by sonication, and the tissue samples were homogenized on ice. Then the samples were centrifuged at 8,000×*g* for 10 min at 4°C, and the supernatants were mixed with the malondialdehyde detection working solution and incubated at 100°C for 60 min. After cooling to room temperature, the mixtures were centrifuged at 10,000×*g* for 10 min, and the supernatants were evaluated using a spectrophotometer (BioTek-Epoch2) at 450, 532, and 600 nm wavelengths. The malondialdehyde content was calculated by the difference of the value at 450, 532, and 600 nm.

For glutathione measurement, the samples were centrifuged at 10,000×*g* for 10 min at 4°C, and the supernatants were subjected to the glutathione assay kit and mixed with the glutathione detection working solution. Then the output was measured immediately at 412 nm by a spectrophotometer (BioTek-Epoch2). The protein concentration of each sample was determined by a bicinchoninic acid protein assay kit (No. 23227; Thermofisher). In addition, malondialdehyde and glutathione levels were normalized according to the protein concentrations.

### Mitochondrial membrane potential

Mitochondrial membrane potential (MMP) was detected with JC-1 staining. When the membrane potential is low, JC-1, as a monomer, emits green excitation light. At higher membrane potentials, JC-1 aggregates increase and emit red light. After treatment with the indicated drugs, cells were incubated with 5 mg/mL JC-1 (No. C2006; Beyotime) for 20 min at 37°C, avoiding light and washed twice with PBS. Then, a fluorescence microscope (Zeiss, Oberkchen, Germany) or FACS flow cytometer (BD Biosciences, Franklin Lakes, NJ, USA) measured the red and green fluorescence cell ratio.

### Scanning and transmission electron microscopy

Fresh colon tissues were washed with cold PBS and fixed with ice-cold 2.5% glutaral overnight. Fixed colon tissues were cut into blocks of 1 mm^3^ thickness. Tissues were fixed with 1% osmium tetroxide for 1 h at room temperature, followed by dehydration using graded ethanol. Tissues were embedded in epoxy resin overnight and sectioned into 100 nm slices. Electron microscopy samples were obtained at the electron microscopy core lab of Sun Yat-Sen University with the scan and transmission electron microscope (JEOL, Japan).

### Intestinal and transepithelial permeability

Intestinal and transepithelial permeability were detected by FITC-dextran (4000 MW; FD4; Sigma Aldrich) as previously described [34]. Mice were administered 0.6 mg/g body weight FD4 in PBS by oral gavage. Blood was collected 4 h later by retro-orbital bleeding. The serum concentration of FITC-dextran was determined using a microplate reader (Infinite 200 pro; TECAN) with an excitation wavelength of 490 nm and an emission wavelength of 530 nm. Serial-diluted FITC-dextran was used to generate a standard curve. The transepithelial permeability was assessed by apical to basolateral FD4 transmission in the transwell plate (Corning 3460; Corning, USA). Briefly, 5 mg/mL FD4 was added to the up-chamber, and after several hours, the sample of the down-chamber was detected by the microplate reader. The transepithelial permeability was presented as the concentration of FD4 in the basolateral chamber.

### MitoTracker staining

After treatment, cells were incubated with 200 nM MitoTracker Red CMXRos (No. C1035; Beyotime) for 30 min at 37°C in the dark. Then the cells were washed with PBS and observed using a laser scanning confocal microscope (Leica, Germany) and a microplate reader.

### Mitochondrial isolation

Mitochondria were extracted using the mitochondrial extraction kit (No. C3601; Beyotime). Cells were rinsed in pre-cold PBS and homogenized in 1 mL ice-cold lysis buffer with a pre-cool Dounce-type glass homogenizer. Then the homogenate was centrifuged 3 times (1000×*g* for 10 min at 4°C) to pellet cell debris and nuclei, and collected the supernatants. Finally, mitochondria from the supernatant were pelleted by centrifugation at 12,000×*g* for 10 min at 4°C.

### Quantification of mitochondrial DNA

Total cellular DNA was extracted using the DNeasy Blood and Tissue kit (No. 51011; Bio-Generating, Changzhou, Jiangsu, China) and quantified using a NanoDrop2000 spectrophotometer (BioTek-Epoch2). A total of 100 ng DNA was amplified using ChamQ SYBR qPCR Master Mix (Vazyme) in a real-time PCR system (Bio-Rad). The target gene content was normalized to β-globin DNA. The primers are listed in Supplementary Table 1.

### Dual-luciferase reporter assay

The Dual-Luciferase Reporter Assay System was used to measure luciferase activity. Transfection efficiency was normalized to Renilla luciferase activity.

### Statistical analysis

Data were presented as means±SD. The statistical significance was analyzed using Student’s *t*-tests or one-way analysis of variance tests (ANOVA), and all tests were two-tailed. The Pearson correlation coefficient was used to estimate the correlation between the mRNA expression levels of MFN2 and ARRB1. The statistical significance was set at *P *<* *0.05.

## Results

### ARRB1 upregulates intestinal barrier function by protecting tight junction during inflammation

**Figure 1.**
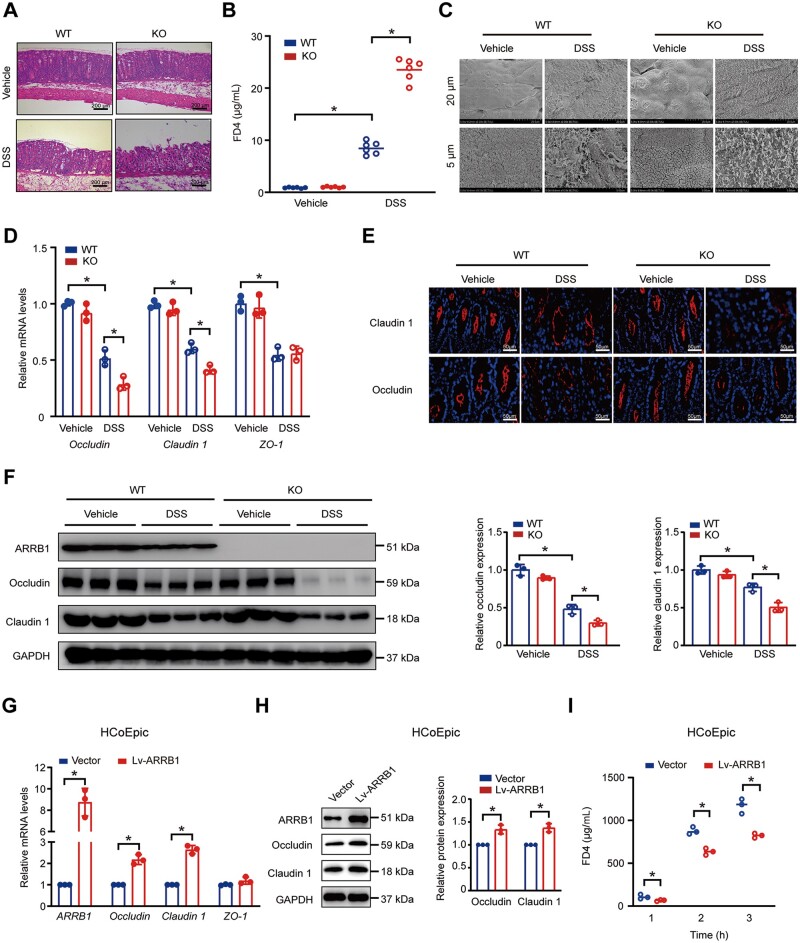
ARRB1 upregulates intestinal barrier function by protecting tight junction during inflammation. WT and *ARRB1* KO mice were fed with or without 3% DSS drinking water for 7 days. (A) Representative histological images of H&E staining (scale = 200 μm). (B) Intestinal permeability was detected by serum FD4 (*n *=* *6). (C) Scanning electron microscopy for the ultrastructure of the tight junction in the intestinal epithelium (scale = 5 and 20 μm). (D) Immuno-fluorescent staining for claudin 1 and occludin. Nuclei were stained with DAPI in blue. Claudin 1 and occludin were visualized in red (scale = 50 μm). (F) Relative mRNA levels of claudin 1, occludin, and ZO-1 by qRT-PCR. (F) Western-blot analysis and densitometric quantification of claudin 1 and occludin expressions. (G) Relative mRNA levels of claudin 1, occludin, and ZO-1 in HcoEpic cells transfected with vector or Lv-ARRB1. (H) Western-blot analysis for claudin 1 and occludin expressions in HcoEpic cells transfected with vector or Lv-ARRB1. (I) The trans-epithelial permeability of HcoEpic cell monolayers was detected by FD4. Data are shown as mean±SD and were analyzed by one-way ANOVA or two-tailed unpaired Student’s *t-*test. *P <* *0.05. WT=wild-type, KO=knockout, DSS=dextran sulfate sodium, FD4=FITC-dextran (4000 MW).

Consistent with our previous study [33], H&E staining showed that DSS induced more colonic mucosal injury in *ARRB1* knockout (KO) mice (Figure 1A). Next, we addressed whether the susceptibility of *ARRB1* deficiency to colitis was due to an intestinal epithelial barrier defect. Intestinal permeability was evaluated by the FD4 test. The serum concentration of FD4 in experimental colitis was significantly higher in *ARRB1* KO mice than in wild-type (WT) mice, indicating that *ARRB1* deficiency contributed to increased intestinal permeability (Figure 1B). The integral membrane components of tight junction proteins regulate the selective permeability between epithelial cells. According to scanning electron microscopy, the tight junction between cells on the apical side of the colonic epithelium was significantly more damaged in *ARRB1* KO mice than in WT mice with colitis (Figure 1C). The mRNA of occludin and claudin 1, two principal components of tight junctions, were reduced more in *ARRB1* KO mice compared with WT mice. The mRNA of ZO-1 was the same reduced whether *ARRB1* deficiency or not (Figure 1D). Immunofluorescence staining showed that claudin 1 and occludin were markedly decreased in *ARRB1* KO mice than in WT mice (Figure 1E). This result was confirmed by Western-blot analysis (Figure 1F). These results indicated that *ARRB1* deficiency impaired the integrity of the intestinal tight junction barrier during colitis. Afterward, we further investigated the potential protective effect of ARRB1 on the tight junction barrier function of HCoEpic cells. Compared with the negative control (Vector), ARRB1 overexpression (Lv-ARRB1) increased the mRNA levels of claudin 1 and occludin but not ZO-1 (Figure 1G). ARRB1 promoted claudin 1 and occludin expression in Western blotting (Figure 1H). In the presence of TNF-α, the concentration of FD4 in the Lv-ARRB1 group increased at a much slower rate, indicating a protective role of ARRB1 on trans-epithelial permeability (Figure 1I). Together, ARRB1 protects the intestinal barrier function by upregulating the expression of tight junction proteins during inflammation.

### ARRB1 deficiency exacerbates mitochondrial dysfunction and oxidative stress in the colonic epithelium of experimental colitis

**Figure 2.**
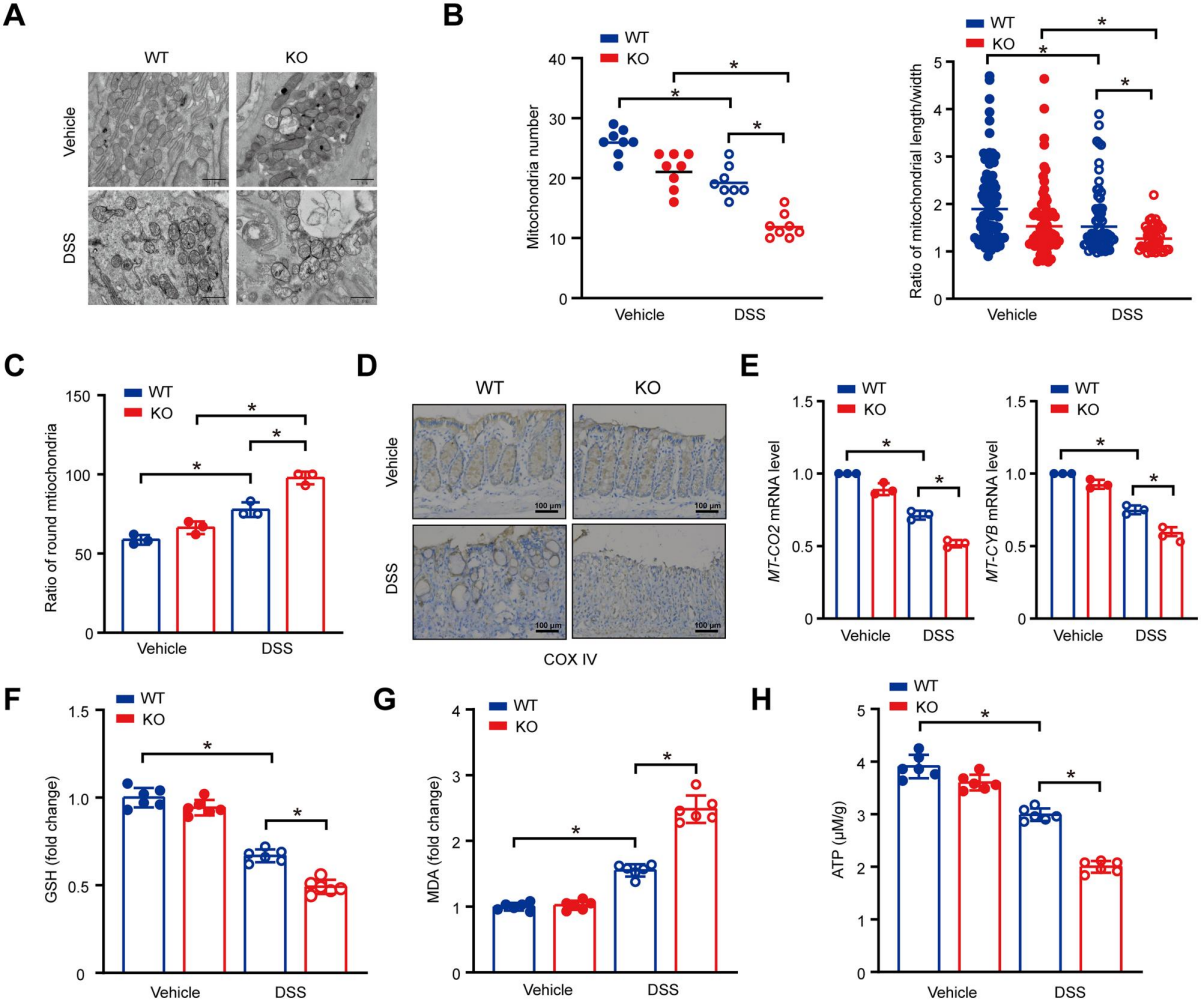
ARRB1 deficiency exacerbates mitochondrial dysfunction and oxidative stress in the colonic epithelium of experimental colitis. (A–F) *ARRB1* WT and KO mice were fed with or without 3% DSS drinking water for 7 days (*n *=* *3–6). (A) Mitochondrial morphology was observed by transmission electron microscopy. (B) Alteration of mitochondrial number and length/width. (C) The ratio of round mitochondria (length/width <2). (D) Immunochemistry staining of COX IV in colon tissue (scale = 100 μm). (E) Relative mRNA expressions of MT-CO2 and MT-CYB. (F) Measurement of glutathione. (G) Relative malondialdehyde levels. (H) Measurement of ATP. Data are shown as mean±SD and were analyzed by one-way ANOVA. *P <* *0.05. WT=wild-type, KO=knockout, DSS=dextran sulfate sodium, GSH=glutathione, MDA=malondialdehyde.

As indicated by swelling of the mitochondria, fracture of the inner or outer membranes, and rupture of the mitochondrial crest, transmission electron microscopy showed that the mitochondria of intestinal epithelial cells from colitis in *ARRB1* KO mice were more severely damaged than those of WT mice in colitis (Figure 2A). Then we analyzed the number, length, and width of mitochondria in a double-blinded manner. There were fewer mitochondria and a higher ratio of round mitochondria (length/width <2) in *ARRB1* KO mice (Figure 2B and C). These results indicated that the *ARRB1* deficiency resulted in a decreased number and severe morphological abnormalities of mitochondria. COX IV, a mitochondrial marker, was reduced more in *ARRB1* KO mice (Figure 2D). The mRNA expressions of mitochondrial cytochrome c oxidase II (MT-CO2) and mitochondrial cytochrome B (MT-CYB), mitochondrial DNA encoded-mitochondrial complexes subunits, were remarkably reduced in *ARRB1* KO mice (Figure 2E). These results indicated that colitis induced more mitochondrial damage in *ARRB1* KO mice. Next, we investigated the effects of ARRB1 on mitochondrial function. In *ARRB1* KO mice, colitis induced significant exhaustion of glutathione and elevation of malondialdehyde, which indicated a deficit in antioxidant capacity (Figure 2F and G). Similarly, ATP production in the colon tissues of *ARRB1* KO mice was significantly lower than that in WT mice (Figure 2H).

### Mitochondrial dysfunction-induced ROS mediates down-regulation of tight junctions *in vitro*

It is unknown whether mitochondrial dysfunction disrupts the intestinal tight junction.

**Figure 3.**
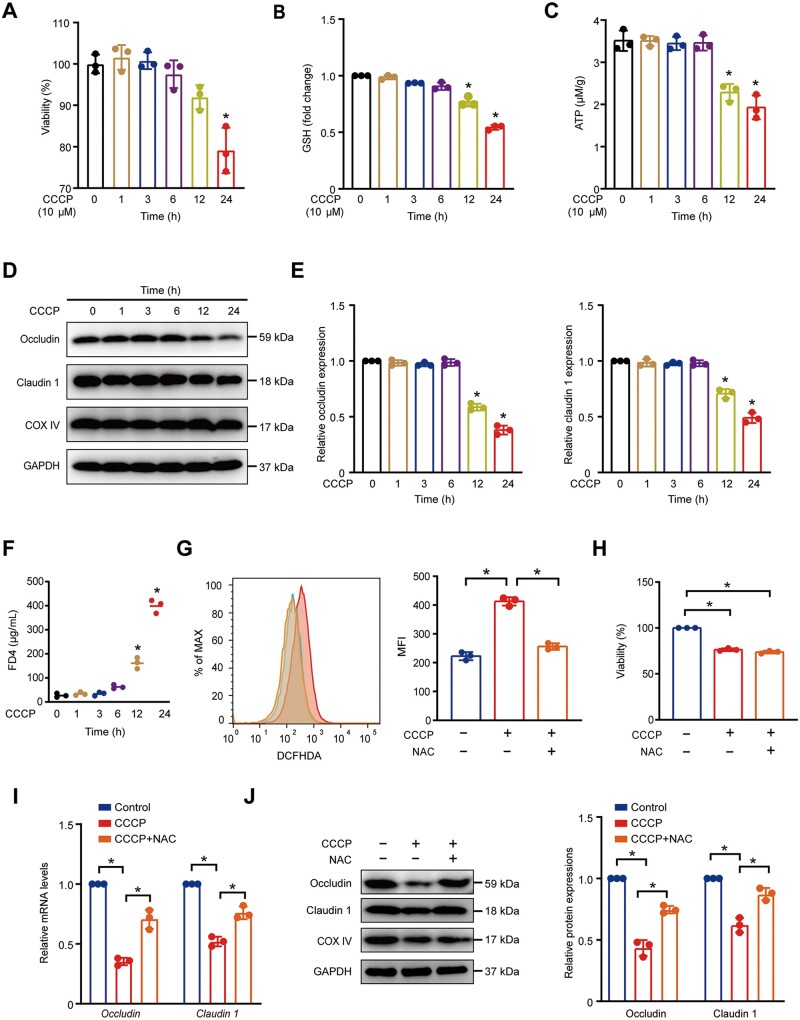
Mitochondrial dysfunction-induced ROS mediates the down-regulation of tight junctions *In vitro*. (A–F) HCoEpic cells were treated with the indicated time of 10 μM CCCP. (A) The cell viability was measured by the CCK-8 assay. (B) Relative levels of glutathione. (C) ATP measurement. (D and E) The expression of claudin 1, occludin, and COX IV by Western-blot analysis. (F) Cellular permeability by FD4 measurement. (G–J) HCoEpic cells were treated with 10 μM CCCP without/and 10 μM NAC for 24 h. (G) Cellular ROS measurement. (H) The cell viability was measured by the CCK-8 assay. (I) Relative mRNA expression of claudin 1, occludin. (J) Western-blot analysis for claudin 1, occludin expressions. Data are shown as mean±SD and were analyzed by one-way ANOVA. *P <* *0.05. CCCP=carbonyl cyanide 3-chlorophenylhydrazone, GSH=glutathione, FD4=FITC-dextran (4000 MW), DCFH-DA=2′-7′dichlorofluorescin diacetate, NAC=N-acetylcysteine, MFI=mean fluorescence intensity.

Next, we investigated the effect of mitochondrial function on tight junctions in HCoEpic cells. We used CCCP, an uncoupler of oxidative phosphorylation, as an inducer of mitochondrial dysfunction [35]. We detected the mitochondrial function and tight junction at different times to explore changes in CCCP-induced mitochondrial dysfunction over time. We found a decrease in the viability of the cells after CCCP treatment for 24h (Figure 3A). Reduced ATP and glutathione levels appeared after CCCP treatment for 12h (Figure 3B and C). The protein expressions of claudin 1 and occludin were reduced after CCCP treatment for 12h (Figure 3D and E). Cellular permeability was also increased by FD4 measurement (Figure 3F). Those results showed that tight junction and cellular permeability were damaged simultaneously with mitochondrial dysfunction but earlier than the decline of cell viability. Mitochondria are the primary source of cellular ROS. NAC, a ROS scavenging agent, significantly reduced cellular ROS accumulation following CCCP treatment (Figure 3G). NAC did not rescue the decreased viability induced by CCCP (Figure 3H). The CCCP-induced downexpression of claudin1 and occludin was reversed by NAC, indicating that ROS was responsible for reducing these proteins induced by mitochondrial dysfunction (Figure 3I and J).

### Knockdown of ARRB1 reduces MMP, antioxidant activity, and ATP production *in vitro*

**Figure 4.**
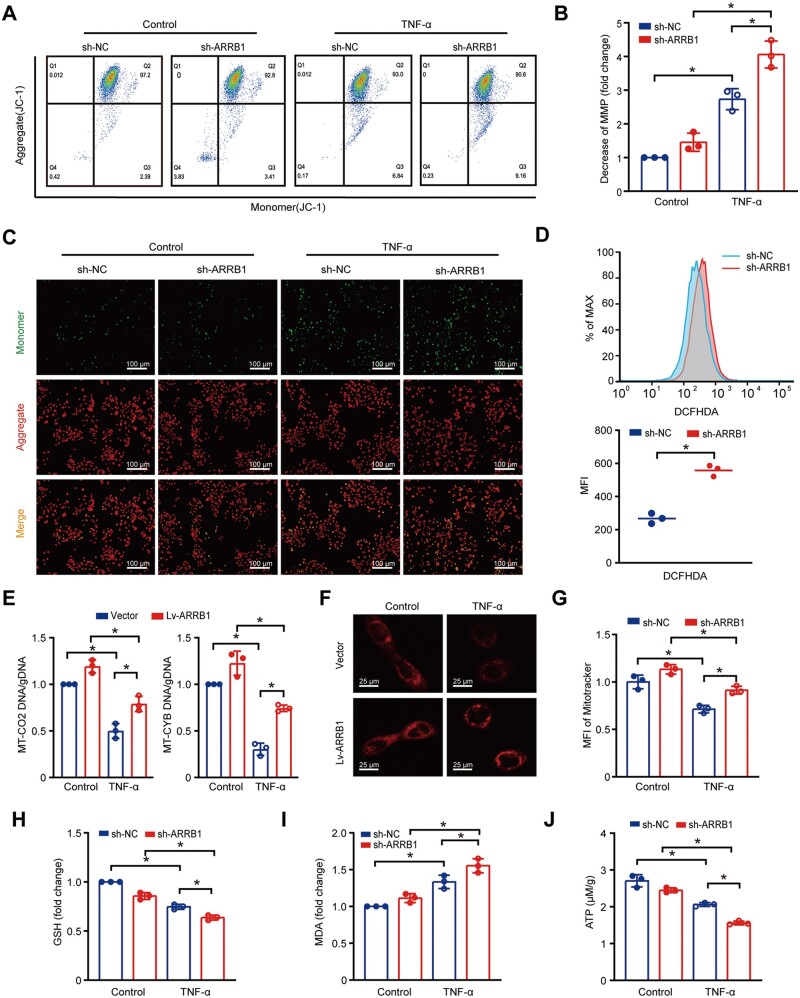
Knockdown of ARRB1 reduces MMP, antioxidant activity, and ATP production *In vitro*. (A) JC-1 staining for MMP by flow cytometry. (B) The relative decrease of MMP. (C) Fluorescence microscopy for JC-1 staining (scale = 100 μm). (D) Cellular ROS measurement of sh-NC and sh-ARRB1 cells by flow cytometry. (E) Relative *MT-CO2* and *MT-CYB* DNA levels in sh-NC and sh-ARRB1 cells after 40 ng/mL TNF-α treatment for 24 h. (F) MitoTracker staining in vector and Lv-ARRB1 cells after 40 ng/mL TNF-α treatment for 24 h by confocal microscopy (scale = 25 μm). (G) The intensity of MitoTracker staining was detected by fluorescence microplate reader in vector and Lv-ARRB1 cells after 40 ng/mL TNF-α treatment for 24 h. (H–J) TNF-α treatment for 24 h in sh-NC and sh-ARRB1 cells. (H) Relative glutathione levels. (I) Relative malondialdehyde levels. (J) ATP production. Data are shown as mean±SD and were analyzed by one-way ANOVA or two-tailed unpaired Student’s *t-*test. *P <* *0.05. MMP=mitochondrial membrane potential, DCFH-DA=2′-7′dichlorofluorescin diacetate, MFI=mean fluorescence intensity, GSH=glutathione, MDA=malondialdehyde.

To clarify the effect of ARRB1 on mitochondrial function *In vitro*, we assessed MMP by measuring the ratio between red and green fluorescence by JC-1 staining. The knockdown of ARRB1 resulted in a more pronounced reduction of MMP in response to TNF-α exposure (Figure 4A and B). Sh-ARRB1 cells consistently exhibited faint red and intense green fluorescence under a fluorescence microscope (Figure 4C). Moreover, the knockdown of *ARRB1* increased cellular ROS accumulation (Figure 4D). As mitochondrial DNA copy numbers can predict the relative number of mitochondria, we extracted it exclusively from the cells and selected MT-CO2 and MT-CYB to represent mitochondrial DNA. We found that TNF-α decreased the levels of MT-CO2 and MT-CYB, but ARRB1 was resistant to this reduction (Figure 4E). ARRB1 delayed TNF-α-induced mitochondrial quantity reduction as assessed by Mito-Tracker Red CMXRos probe staining (Figure 4F and G). The knockdown of *ARRB1* reduced antioxidant activity with a significant decrease in glutathione and an evaluation of malondialdehyde level (Figure 4H and I). The production of ATP was also reduced in sh-ARRB1 cells (Figure 4J). Thus, these results suggest that *ARRB1* knockdown reduces MMP and disrupts mitochondrial function *in vitro*.

### ARRB1 preserves mitochondrial function through promoting PINK1/parkin-dependent mitophagy

**Figure 5.**
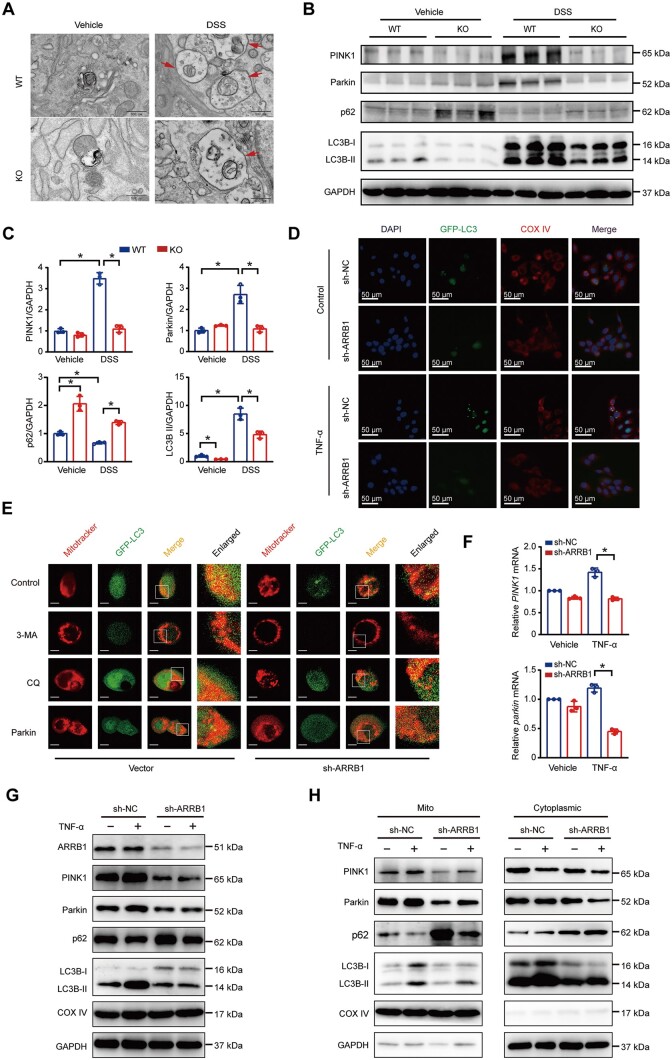
ARRB1 preserves mitochondrial function through promoting PINK1/parkin-dependent mitophagy. (A–C) *ARRB1* WT and KO mice were fed with or without 3% DSS drinking water (*n *=* *3–6). (A) Mitochondrial autophagosomes (arrow) were observed by TEM. (B and C) Western-blot analysis and densitometric quantification for mitophagy-associated protein expressions (PINK1, Parkin, p62, LC3B). (D) Co-localization immune-fluorescent staining of GFP-LC3 and COX IV after TNF-α treatment in sh-NC and sh-ARRB1 cells for 24 h. (scale = 50 μm). (E) Co-localization of GFP-LC3 and mitotracker after 3-MA, CQ treatment, and parkin transfection in sh-NC and sh-ARRB1 cells by confocal microscopy. (scale = 25 μm). (F) Relative mRNA levels of pink1 and parkin. (G) PINK1, parkin, and LC3B expression in the whole-cell lysis. (H) PINK1, parkin, and LC3B expression in the mitochondrial and mito-depleted cytoplasm fragment. Data are shown as mean±SD and were analyzed by one-way ANOVA or two-tailed unpaired Student’s *t*-test. *P <* *0.05. WT=wild-type, KO=knockout, DSS=dextran sulfate sodium, 3-MA=3-methyladenine, CQ=chloroquine, Mito=mitochondria.

Mitophagy defects lead to damaged mitochondria accumulation and pathological change. PINK1/Parkin-mediated mitophagy is a significant pathway of mitophagy. Having demonstrated that the absence of ARRB1 disrupted mitochondrial function, we hypothesized that ARRB1 preserved mitochondrial quantity and function through PINK1/parkin-mediated mitophagy. Transmission electron microscopy showed an increase in mitochondrial auto-phagosomes in DSS-induced colitis. *ARRB1* KO mice exhibited fewer mitochondrial auto-phagosomes than WT mice (Figure 5A). Western blotting showed that the mitophagy-associated proteins PINK1, Parkin, and LC3B were significantly upregulated in experimental colitis, but *ARRB1* KO inhibited the upregulation of these proteins (Figure 5B and C). These results demonstrated that the deletion of *ARRB1* inhibited mitophagy, which is activated to clear damaged mitochondria in colitis. Next, we examined the effect of ARRB1 on the mitophagic flux in HCoEpic cells. The co-localization of LC3 with COX IV, an indicative mitophagy marker, was less prominent in sh-ARRB1 cells than in sh-NC cells (Figure 5D). We analyzed the colocalization of LC3 and mitotracker. CQ treatment increased the colocalization of LC3 and mitotracker because CQ blocks autophagosome degradation. We found that mitophagy was rescued after transfecting parkin to ARRB1 KO cells (Figure 5E). *ARRB1* knockdown significantly downregulated the mRNA expressions of PINK1 and Parkin (Figure 5F). TNF-α increased the expressions of PINK1, Parkin, and LC3B-II. However, the knockdown of *ARRB1* suppressed these up-expressions (Figure 5G). We isolated mitochondria to confirm that these mitophagy-associated proteins were changed specifically in mitochondria. TNF-α enriched PINK1, Parkin, and LC3B-II in the mitochondrial fraction, but this effect was suppressed in *sh-ARRB1* cells (Figure 5H). Taken together, ARRB1 promotes PINK1/parkin-mediated mitophagy.

### Inhibition of mitophagy downregulates ARRB1-mediated tight junction protection

**Figure 6.**
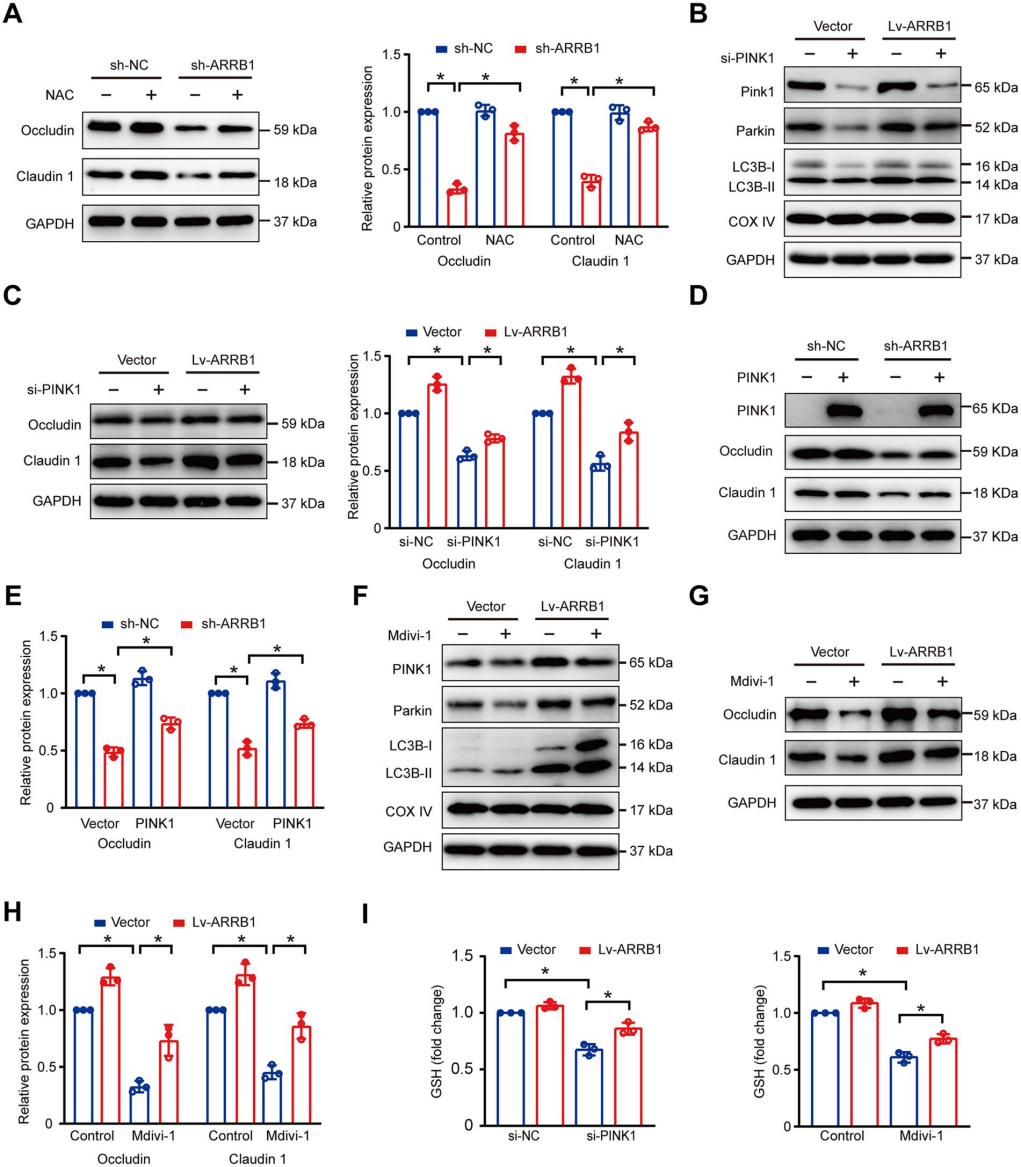
Inhibition of mitophagy downregulates ARRB1-mediated tight junction protection. (A) Claudin 1 and occludin expression in sh-NC and sh-ARRB1 cells with or without 10 μM NAC treatment for 24 h. (B and C) si-NC or si-PINK1 transfected in Vector and Lv-ARRB1 cells. (B) PINK1, Parkin, and LC3B expressions by Western-blot analysis. (C) Claudin 1 and occludin expressions by Western-blot analysis. (D and E) Claudin 1 and occludin expressions after PINK1 transfection into sh-NC and sh-ARRB1 cells. (F–H) Control or 10 μM Mdivi-1 treatment in vector and Lv-ARRB1 cells. (F) PINK1, Parkin, and LC3B expressions by Western-blot analysis. (G and H) Claudin 1 and occludin expressions by Western-blot analysis. (I) Relative glutathione levels in Vector and LvARRB1 cells with or without PINK1 siRNA/10 μM Mdivi-1 treatment. Data are shown as mean±SD and were analyzed by one-way ANOVA or two-tailed unpaired Student’s *t*-test. *P <* *0.05. NAC=N-acetylcysteine, Mdivi-1=mitochondrial division inhibitor 1, GSH=glutathione.

Subsequently, we validated the importance of dysregulated mitophagy on intestinal tight junctions by *ARRB1* knockdown. The silencing of ARRB1 downregulated the expression of claudin 1 and occludin, and NAC reversed this downexpression (Figure 6A). Thus, the reduction of tight junction proteins by *ARRB1* knockdown was due to ROS accumulation. Then, we used PINK1 siRNA to downregulate expressions of PINK1. In addition, the downregulation of PINK1, Parkin, and LC3B-II indicated that si-PINK1 inhibited mitophagy, undermining ARRB1-mediated claudin 1 and occludin up-regulations (Figure 6B and C). Additionally, transfecting with PINK1 plasmid to sh-ARRB1 cells, we found that claudin 1 and occludin expressions were restored (Figure 6D and E). Moreover, we used Mdivi-1 as a mitophagy inhibitor. Mdivi-1 inhibited mitophagy and reduced the expressions of claudin 1 and occludin (Figure 6F–H). Furthermore, the inhibition of mitophagy medicated by si-PINK1 and Mdivi-1 significantly reduced glutathione levels (Figure 6I).

### ARRB1 protects tight junction through MFN2-mediated mitophagy

**Figure 7.**
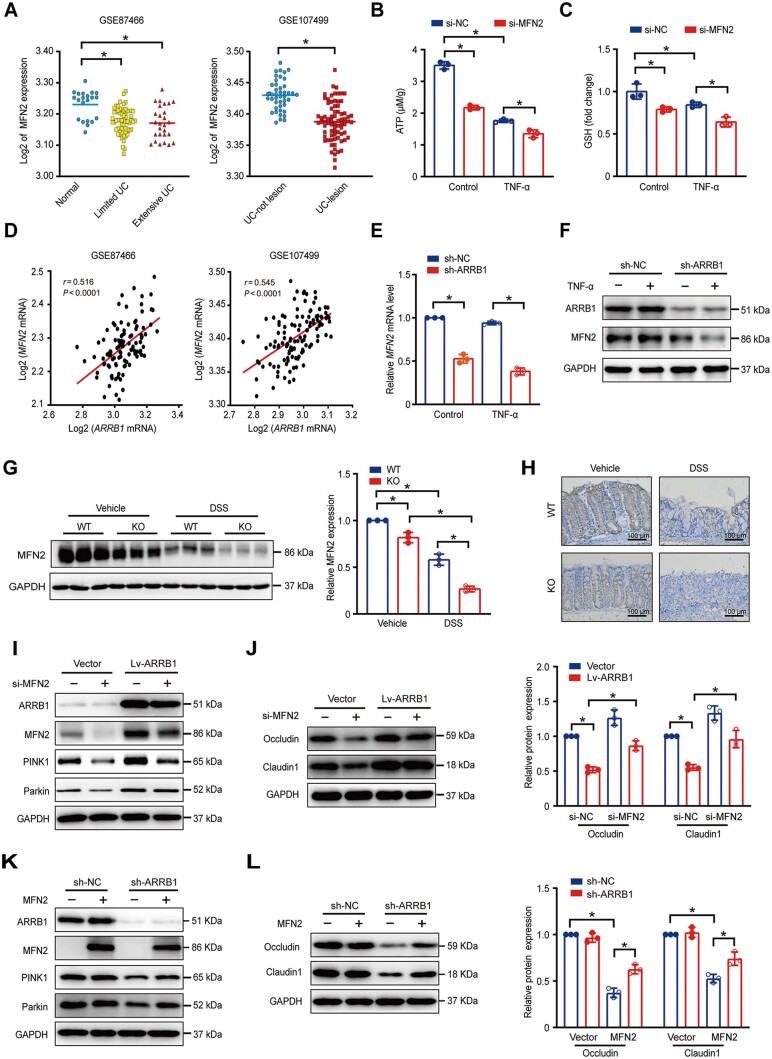
ARRB1 protects tight junction through MFN2-mediated mitophagy. (A) Relative MFN2 mRNA expression was analyzed by two GEO data (GSE87466 and GSE 107499). (B) ATP production in si-NC or si-MFN2 cells with or without TNF-α treatment. (C) Relative glutathione levels in si-NC or si-MFN2 cells with or without TNF-α treatment. (D) Correlation between MFN2 mRNA with arrb1 mRNA analyzed by GEO data (GSE87466 and GSE 107499). (E) MFN2 mRNA expression in sh-NC and sh-ARRB1 cells with or without TNF-α treatment for 24 h. (F) MFN2 expression in sh-NC and sh-ARRB1 cells with or without TNF-α treatment for 24 h. (G and H) *ARRB1* WT and KO mice were fed with or without 3% DSS drinking water (*n *=* *3–6). (G) MFN2 expression by Western-blot analysis. (H) Immunochemistry staining of MFN2 in colon tissues. (I and J) si-NC or si-MFN2 transfected in vector and Lv-ARRB1 cells. (I) MFN2, PINK1, and parkin expression by Western-blot analysis. (J) Claudin 1 and occludin expression by Western-blot analysis. (K and L) MFN2 plasmid transfection into sh-NC and sh-ARRB1 cells. (K) MFN2, PINK1, and parkin expression by Western-blot analysis. (L) Claudin 1 and occludin expression by Western-blot analysis. Data are shown as mean±SD and were analyzed by one-way ANOVA or two-tailed unpaired Student’s *t*-test. The correlation between mRNA expression levels of MFN2 and ARRB1 was analyzed by the Pearson correlation coefficient. *P <* *0.05. UC=ulcerative colitis, DSS=dextran sulfate sodium, GSH=glutathione, WT=wild-type, KO=knockout.

Next, we intended to investigate the mechanisms by which ARRB1 modulated mitophagy. MFN2 located on the outer mitochondrial membrane, is not only the regulator of mitochondrial fusion but also involved in the progression of mitophagy. By analyzing two GEO data (GSE87466 and GSE 107499), we discovered that MFN2 was significantly down-regulated in UC (Figure 7A). Then, we found that silencing MFN2 with siRNA reduced ATP production and glutathione levels, indicating that MFN2 deficiency contributed to mitochondrial dysfunction **(**Figure 7B and C).

We analyzed GSE87466 and GSE107499 data and revealed a significant positive correlation between MFN2 and ARRB1 mRNA (Figure 7D). Thus, we hypothesized that ARRB1 regulated mitophagy via MFN2. We demonstrated that the knockdown of *ARRB1* reduced MFN2 mRNA and protein expression (Figure 7E and F). Subsequently, we detected MFN2 expression in colonic tissue. MFN2 expression was reduced in colitis, and ARRB1 deficiency exacerbated this decrease (Figure 7G). Immunohistochemistry presented consistent results (Figure 7H).

Furthermore, the knockdown of MFN2 abrogated the ARRB1-mediated mitophagy activation (Figure 7I) and claudin 1, occludin upexpression (Figure 7J). Transfecting with MFN2 plasmid to sh-ARRB1 cells, we found that mitophagic proteins, claudin 1, and occludin expressions were restored (Figure 7K and L). These results indicate that MFN2 is involved in ARRB1-upregulated mitophagy.

### Interaction of ARRB1 with E2F1 enhances MFN2 transcription

**Figure 8.**
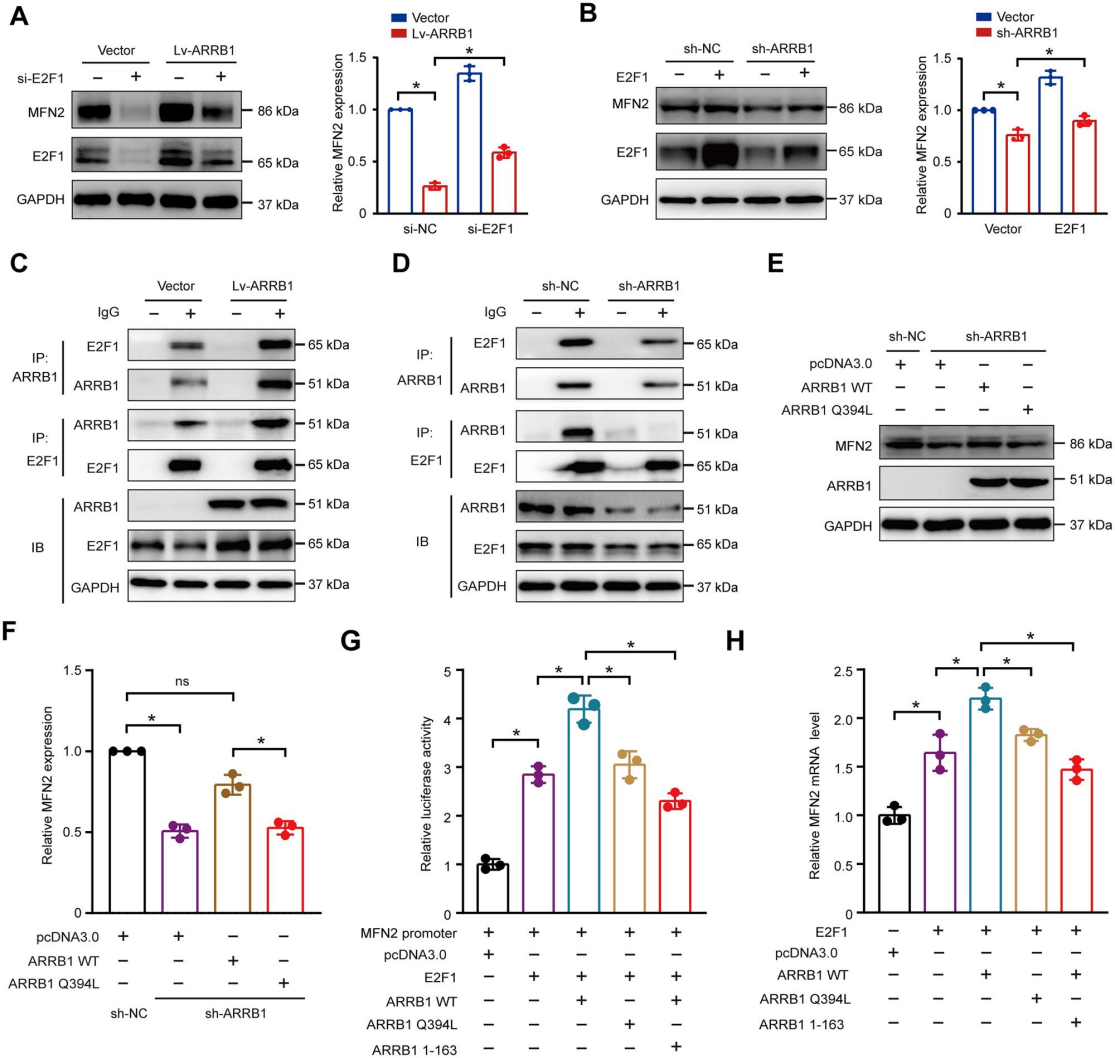
Interaction of ARRB1 with E2F1 enhances MFN2 transcription. (A) MFN2 expression in vector and LvARRB1 cells transfected with or without E2F1 siRNA. (B) MFN2 expression in sh-NC and sh-ARRB1 cells transfected with or without E2F1 plasmid. (C and D) The interaction between ARRB1 and E2F1 was detected by co-immunoprecipitation. (E and F) MFN2 expression in sh-NC and sh-ARRB1 cells transfected with pcDNA3.0, ARRB1 WT, or ARRB1 mutant Q394L plasmid. (G) The relative luciferase activity of MFN2 transfected with pcDNA3.0, ARRB1 WT, ARRB1 mutant Q394L, or ARRB1 1–163 plasmid. (H) Relative MFN2 mRNA expression transfected with pcDNA3.0, ARRB1 WT, ARRB1 mutant Q394L, or ARRB1 1–163 plasmid. Data are shown as mean±SD and were analyzed by one-way ANOVA or two-tailed unpaired Student’s *t*-test. *P <* *0.05.

We then investigated how ARRB1 regulated MFN2 expression. It is known that ARRB1 translocates from the cytoplasm to the nucleus, interacting with transcription co-factors such as p300 and NF-kappaB to modulate targeted gene transcription [26, 27]. A previous study demonstrated that E2F1 activates MFN2 expression by binding to its promoter [36]. Consistent with this finding, we found that the knockdown of E2F1 inhibited ARRB1-mediated MFN2 upexpression (Figure 8A), and the transfection of E2F1 restored the repression of MFN2 by the knockdown of ARRB1 (Figure 8B). Co-immunoprecipitation showed an interaction between ARRB1 and E2F1 (Figure 8C and D). These results indicated that ARRB1 might regulate MFN2 expression by interaction with E2F1. However, E2F1 worked as a transcriptional factor in the nucleus. To identify whether nuclear translocation of ARRB1 regulates the expression of MFN2, we performed transient transfection with ARRB1 mutant Q394L, in which glutamine 394 has been mutated to leucine to create a nuclear export signal [37]. The ARRB1 mutant Q394L failed to rescue MFN2 expression in sh-ARRB1 cells, suggesting that nuclear translocation of ARRB1 was required for transcriptional regulation of MFN2 (Figure 8E and F). The Luciferase activity assay also showed that the mutant Q394L did not enhance E2F1-mediated transcription from the MFN2 promoter (Figure 8G). Previous studies have shown that amino acids 1–163 of ARRB1 are required to bind to E2F1 [38]. Thus, ARRB1 1–163 could competitively inhibit the interaction of ARRB1 WT and E2F1. The luciferase activity assay showed that ARRB1 1–163 inhibited the ARRB1-mediated MFN2 upexpression by competitive binding to E2F1 (Figure 8G). Consistent with the luciferase activity assay, ARRB1 WT increased the MFN2 mRNA levels, but not ARRB1 mutant Q394L. Moreover, the ARRB1-mediated upregulation of MFN2 was inhibited by ARRB1 1–163 (Figure 8H). As a result, ARRB1-E2F1 interaction enhanced MFN2 transcription.

## Discussion

ARRB1 was initially discovered and described as a negative regulator of G protein-dependent signaling in the GPCR signaling pathway [23]. Further research has found that ARRB1, as a scaffolding protein, interacts with several effector proteins [39]. ARRB1 regulates fundamental biological processes such as proliferation and apoptosis [40]. Our previous study found that ARRB1 is involved in COX-1/PGE2/EP4-mediated mucosal protection in colitis [33]. However, the effect of ARRB1 on colitis remains unclear. Lee *et al*. [31] initially found that the deficiency of ARRB1 protects against experimental colitis, then found that nonhematopoietic ARRB1 confers protection against experimental colitis [32]. ARRB1-dependent signaling in hematopoietic and nonhematopoietic cells differentially regulates colitis pathogenesis. Due to the contribution of intestinal epithelial barrier impairment to the initiation and progression of IBD, we sought to investigate the role of ARRB1 in the intestinal barrier. ARRB1 deficiency reduced the expression of claudin 1 and occludin, which are essential components of the intestinal tight junctions barrier. ARRB1 protects the tight junction of the intestinal epithelium to maintain the function of the intestinal barrier. As a crucial component of the physical barrier, the tight junction between intestinal epithelial cells regulates paracellular permeability. Emerge evidence *in vivo* and *In vitro* has shown decreased expression of tight junctions and increased intestinal permeability due to reduced epithelial barrier function [41]. Moreover, maintaining the integrity and function of the intestinal epithelial barrier may contribute to preventing and treating IBD [42]. Therefore, ARRB1 is critical in maintaining intestinal tight junction barrier function.

Recent evidence has placed mitochondria as the gatekeeper of intestinal epithelial cell homeostasis [43]. Mitochondria supply energy and essential metabolites for cell activities. It has been shown that dysregulated mitochondrial signaling and function contribute to the pathogenesis of IBD [44]. Two recent studies proved that Paneth cells are highly susceptible to mitochondrial dysfunction in IBD. Prohibitin, a substantial component protein of the inner mitochondrial membrane, is crucial for the optimal assembly and function of the respiratory chain. Mice lacking prohibitin in intestinal epithelial cells developed Paneth cell abnormalities and spontaneous ileitis preceded by mitochondrial dysfunction [45]. In another study, Khaloian *et al.* [46] demonstrated that inflammation-associated mitochondrial dysfunction in the intestinal epithelium triggered a metabolic imbalance and drove intestinal stem cells to transition into aberrant Paneth cells. Zhang *et al*. [47] found that knockdown of *ARRB1* inhibited isoproterenol-induced mitochondrial ROS production. High glucose induces mitochondrial dysfunction by inhibiting ARRB signaling to attenuate the cardio-protective effects of glucagon-like peptide 1 [29]. However, some studies have found the opposite result. The deletion of ARRB1 did not affect cerebral ischemia-induced inflammation and oxidative stress but markedly suppressed autophagy and induced neuronal apoptosis/necrosis [48].

Recent research has demonstrated the association between ARRB1 and autophagy. ARRB1 regulates BECN-dependent auto-phagosome formation to mediate neuroprotection in cerebral ischemia [48]. Lei *et al.* [49] indicated that HBx-induced hepatocellular carcinogenesis through ARRB1-mediated autophagy. Mitophagy is protective in eliminating dysfunctional or redundant mitochondria to maintain mitochondrial homeostasis [50]. Mitophagy impairment perturbs mitochondrial function and causes progressive accumulation of defective organelles, leading to cell and tissue damage [51]. Mitophagy plays a vital role in intestinal inflammation. Vincent *et al.* [52] found that NIX deficiency aggravated colitis via mitophagic inhibition, which induced the inability to clear damaged or dysfunctional mitochondria. Consistently, we found mitophagy was involved in ARRB1-mediated tight junction protection. ARRB1 deficiency suppressed PINK1-parkin-dependent mitophagy and induced severe oxidative stress and ROS accumulation. Mitofusins are preferred targets at the OMM and are ubiquitylated by E3 ligases [53]. MFN2 is a regulator of mitochondrial fusion and is associated with mitophagy. Upon mitophagy induction, ubiquitination of MFN2 targets them for degradation by proteasomes, quickly leading to the abrogation of mitochondrial fusion events and resulting in mitochondrial fragmentation. Thus, MFN2 acts as a pro-mitophagic receptor. It has been demonstrated that loss of MFN2 is associated with defects in autophagosome or autophagosome-lysosome formation, two events of mandatory nature for mitophagy [54]. Consistently, depletion of MFN2 in murine cardiomyocytes caused an accumulation of defective mitochondria. MFN2 deficiency inhibited mitophagy and increased apoptosis [55]. MFN2-induced mitophagy improved disease prognosis in gastric cancer [56]. In this work, we found that ARRB1 inhibited mitophagy through decreasing MFN2 expression. Down-expression of MFN2 leads to suppressing PINK1/parkin-dependent mitophagy, subsequently hindering the elimination of dysfunctional mitochondria. Furthermore, ARRB1 regulates MFN2 transcriptionally. ARRB1 is a scaffold protein in multiple signaling pathways and co-factors gene transcription regulation. Zecchini *et al.* [57] demonstrated that nuclear ARRB1-induced pseudohypoxia and cellular metabolism reprogramming in prostate cancer via regulation of HIFA transcription activity. Another study indicated that ARRB1 is bound to E2F1 target genes that modulate epithelial–mesenchymal transition in nicotine-induced growth of lung tumors [37]. This study demonstrated that ARRB1 regulates MFN2 transcription by cooperating with E2F1. This finding explains how ARRB1 functions as a transcriptional regulator in cell growth, apoptosis, and mitochondrial function modulation.

In conclusion, the KO of ARRB1 resulted in mitochondrial dysfunction and a disrupted intestinal barrier in colitis. Mechanistically, ARRB1 promoted PINK1/parkin-mediated mitophagy to eliminate damaged mitochondria. Silencing of ARRB1 resulted in ROS accumulation and exacerbated oxidative stress. As a transcriptional regulator, ARRB1 interacted with E2F1 to promote MFN2 transcription and enhance cell mitophagy flux. Our findings may open new therapeutic avenues for maintaining mitochondrial dysfunction to treat colitis.

## Supplementary Material

goae084_Supplementary_Data
