# Supplementary material for: β-arrestin1 protects intestinal tight junction through promoting mitofusin 2 transcription to drive parkin-dependent mitophagy in colitis
Source: Gastroenterol Rep (Oxf). 2024 Sep 6;12:goae084. doi: 10.1093/gastro/goae084 (PMC11379473; doi:10.1093/gastro/goae084)
Supplement: goae084_Supplementary_Data [file goae084_supplementary_data.docx]

| **Gene** | **Species** | **Primer** | **Sequence** |
| --- | --- | --- | --- |
| ***CLDN1*** | mouse | Forward primer  Reverse primer | 5’ GGGGACAACATCGTGACCG 3’  5’ AGGAGTCGAAGACTTTGCACT3’ |
| ***OCLN*** | mouse | Forward primer  Reverse primer | 5’ TTGAAAGTCCACCTCCTTACAGA 3’  5’ CCGGATAAAAAGAGTACGCTGG 3’ |
| ***PINK1*** | mouse | Forward primer  Reverse primer | 5’ TTGCCCCACACCCTAACATC 3’  5’ GCAGGGTACAGGGGTAGTTCT 3’ |
| ***PARK2*** | mouse | Forward primer  Reverse primer | 5’ TTGCTGGGACGATGTCTTAATTC 3’  5’ AAAGCTACCGACGTGTCCTTG 3’ |
| ***MFN2*** | mouse | Forward primer  Reverse primer | 5’ TGCACCGCCATATAGAGGAAG 3’  5’ TCTGCAGTGAACTGGCAATG 3’ |
| ***ARRB1*** | mouse | Forward primer  Reverse primer | 5’ CCGAGGACAAGAAGCCACTGA 3’  5’ AGAGTGACTGAGCATGGAAGGT 3’ |
| ***MT-CO2*** | mouse | Forward primer  Reverse primer | 5’ ATAATCCCAACAAACGACCT3’  5’ CTCGGTTATCAACTTCTAGCA3’ |
| ***MT-CYB*** | mouse | Forward primer  Reverse primer | 5’ TATACACGCAAACGGAGCCT 3’  5’ CCTCGTCCGACATGAAGGAA 3’ |
| ***ACTB*** | mouse | Forward primer  Reverse primer | 5’ AGAGGGAAATCGTGCGTGAC 3’  5’ CAGGAAGGAAGGCTGGAAGAG 3’ |
| ***CLDN1*** | Human | Forward primer  Reverse primer | 5’GCAACTCTGGATGGGATTGC 3’  5’TTGCAGCAGCGAAGTGTTTC 3’ |
| ***OCLN*** | Human | Forward primer  Reverse primer | 5’CGGCGAGTCCTGTGATGAG 3’  5’TCTTGTATTCCTGTAGGCCAGT 3’ |
| ***PINK1*** | Human | Forward primer  Reverse primer | 5’ TGTGGAACATCTCGGCAGG 3’  5’ CGGAGAACCCGGATGATGTT 3’ |
| ***PARK2*** | Human | Forward primer  Reverse primer | 5’ GTGTTTGTCAGGTTCAACTCCA 3’  5’ GAAAATCACACGCAACTGGTC 3’ |
| ***MFN2*** | Human | Forward primer  Reverse primer | 5’ CCCCCTTGTCTTTATGCTGATGTT 3’  5’ TTTTGGGAGAGGTGTTGCTTATTTC 3’ |
| ***ARRB1*** | Human | Forward primer  Reverse primer | 5’ GCGAGCACGCTTACCCTTT 3’  5’ CAAGCCTTCCCCGTGTCTTC 3’ |
| ***MT-CO2*** | Human | Forward primer  Reverse primer | 5’ CGCATCCTTTACATAACAGACG’  5’ TAGGAGTTGAAGATTAGTCCGC’ |
| ***MT-CYB*** | Human | Forward primer  Reverse primer | 5’ GAATCACCTCCCATTCCGAT 3’  5’ AGGAAATATCATTCGGGCTT 3’ |
| ***ACTB*** | Human | Forward primer  Reverse primer | 5’ GTCTTCCCCTCCATCGTG 3’  5’ AGGGTGAGGATGCCTCTCTT 3’ |
| ***β-globin DNA*** | Human | Forward primer  Reverse primer | 5’ CAGGAGCTGTGGGAGGAAGATAA 3’  5’ TTATGGTTGGGATAAGGCTGGATT 3’ |
| ***MT-CO2 DNA*** | Human | Forward primer  Reverse primer | 5’ GCTGTCCCCACATTAGGCTT 3’  5’ CGATGGGCATGAAACTGTGG 3’ |
| ***MT-CYB DNA*** | Human | Forward primer  Reverse primer | 5’ CCCATCCAACATCTCCGCAT 3’  5’ GAGGCGTCTGGTGAGTAGTG 3’ |
